# Circulating microRNA Related to Cardiometabolic Risk Factors for Metabolic Syndrome: A Systematic Review

**DOI:** 10.3390/metabo12111044

**Published:** 2022-10-30

**Authors:** Paula N. Brandão-Lima, Gabrielli B. de Carvalho, Tanyara B. Payolla, Flavia M. Sarti, Marcelo M. Rogero

**Affiliations:** 1 Department of Nutrition, School of Public Health, University of Sao Paulo, 715 Dr Arnaldo Avenue, Pacaembu, Sao Paulo 01246-904, SP, Brazil; 2 School of Arts, Sciences and Humanities, University of Sao Paulo, 1000 Arlindo Bettio Avenue, Sao Paulo 03828-000, SP, Brazil

**Keywords:** miRNA, serum, plasma, obesity, type 2 diabetes, metabolic syndrome

## Abstract

MicroRNA regulates multiple pathways in inflammatory response, adipogenesis, and glucose and lipid metabolism, which are involved in metabolic syndrome (MetS). Thus, this systematic review aimed at synthesizing the evidence on the relationships between circulating microRNA and risk factors for MetS. The systematic review was registered in the PROSPERO database (CRD42020168100) and included 24 case-control studies evaluating microRNA expression in serum/plasma of individuals ≥5 years old. Most of the studies focused on 13 microRNAs with higher frequency and there were robust connections between miR-146a and miR-122 with risk factors for MetS, based on average weighted degree. In addition, there was an association of miR-222 with adiposity, lipid metabolism, glycemic metabolism, and chronic inflammation and an association of miR-126, miR-221, and miR-423 with adiposity, lipid, and glycemic metabolism. A major part of circulating microRNA was upregulated in individuals with risk factors for MetS, showing correlations with glycemic and lipid markers and body adiposity. Circulating microRNA showed distinct expression profiles according to the clinical condition of individuals, being particularly linked with increased body fat. However, the exploration of factors associated with variations in microRNA expression was limited by the variety of microRNAs investigated by risk factor in diverse studies identified in this systematic review.

## 1. Introduction

Dyslipidemia, high blood pressure, hyperglycemia, insulin resistance, and obesity are cardiometabolic risk factors associated with metabolic syndrome (MetS) and noncommunicable diseases (NCD) [1,2,3,4]. MetS is low-grade systemic inflammation, or meta inflammation, meaning it comprises a metabolically triggered inflammation process [2,4,5]. This condition is related to the expansion of white adipose tissue, consisting of hypertrophy and hyperplasia of adipocytes [2,4,5]. Exposure of adipocytes to oxidative stress and overexpression of inflammatory cytokines induce cellular responses, mediated by cellular kinases, including JNK and IKK, which are related to the inactivation of the insulin receptor substrate, resulting in impaired insulin action and sensitivity, an important risk factor for MetS [5].

MicroRNA corresponds to small non-coding RNA molecules (21–23 nucleotides) that regulate numerous processes related to metabolic diseases [6]. MicroRNA mediates gene expression through post-transcriptional mechanisms [6]. Circulating microRNAs are present in extracellular fluids (e.g., plasma) and tissues and may be secreted in vesicles (exosomes, microvesicles, and apoptotic bodies) or combined with proteins (argonauts—AGO, low- and high-density lipoproteins—LDL and HDL—and nucleophosmin 1—NPM1) [7,8]. These small RNA molecules are potential biomarkers for cardiometabolic diseases and may comprise markers for investigation of NCD early risk [9,10].

Recent studies have focused on the relationship of circulating microRNA in relation to different metabolic conditions, especially obesity [11,12,13,14,15], type 2 diabetes (T2D) [15,16,17,18], MetS [9,19,20], and hypertension [15,21,22], which are major risk factors for the development of systemic complications [15]. In this context, evidence showed an association between miR-122 expression and increased risk for MetS and T2D [23,24]; higher plasma expression of miR-222 and fasting glucose and %HbA1c in individuals with T2D [25]; and potential use of miR-130b as a biomarker for obesity [9,10].

Considering the scarcity of evidence on the link between MetS and circulating microRNA, the investigation of connections between microRNA and diabetes, hypertension, dyslipidemia, and obesity may shed light on pathways related to MetS. In addition, circulating microRNA may present diverse patterns during various life stages; therefore, their connections with health conditions related to MetS may require different interpretations. Thus, the systematic review aimed to identify and integrate the evidence on the relationships between circulating microRNA and main risk factors for MetS in diverse life stages, highlighting the potential use of microRNA as MetS biomarkers.

## 2. Materials and Methods

The systematic review was prepared according to the Preferred Reporting Items for Systematic Reviews and Meta-Analyses (PRISMA) [26], supplemented by the reporting of Meta-analyses of Observational Studies in Epidemiology (MOOSE) [27]. Considering that the analysis was based on evidence published in scientific studies without possibility of identification of subjects, the requirements were waived for ethical approval by Research Ethics Committee and application of informed consent. The study protocol was registered in the International Prospective Register of Systematic Reviews (PROSPERO) database (registration number CRD42020168100).

### 2.1. Search Strategy

The literature search was performed in March 2020 on Lilacs, PUBMED/Medline, Embase, Web of Science, Scopus, and ClinicalTrials.gov, and gray literature on Google Scholar. Keywords related to glycemic and lipid metabolism, inflammation, and overweight/obesity were combined using Boolean terms in the search (Supplementary Table S1). In addition, the authors performed manual search of additional studies in the references cited in eligible studies. The search strategy was performed without restriction referring to publication date or language. However, one article published in Chinese was excluded from the analysis due to limitations identified in the translation from Chinese to English.

### 2.2. Eligibility Criteria

The present systematic review focused on cross-sectional studies investigating MetS factors (diabetes, obesity, dyslipidemia, hypertension, and/or dyslipidemia), published in full version and including case and control groups. Considering that individuals under 5 years old should experience important developmental milestones that potentially affect microRNA expression, only studies including individuals ≥5 years old were included, evaluating the relationship between microRNA levels in serum/plasma and biomarkers related to glycemic and/or lipid metabolism, inflammation, and/or anthropometric variables. Eligible studies should include a control group comprising individuals without the clinical condition under investigation in the case group (healthy vs. unhealthy group). In addition, studies should include only individuals with one disease or metabolic complication (i.e., studies including individuals with multiple conditions were excluded from the analysis).

In order to minimize wide variation in circulating microRNA expression, the following studies were considered ineligible: studies focusing on the analysis of microRNA in saliva/vesicles/blood cells or including individuals with clinical complications, such as cancer, kidney disease, thyroid dysfunction, AIDS, or acute inflammatory processes.

### 2.3. Study Selection and Data Extraction

Three researchers (PNBL, GBC, TBP) conducted the literature search and selection stages independently: first, studies identified in the search were screened by title and abstract; in sequence, full papers of studies selected in the first stage were analyzed to check eligibility (Supplementary Table S2). Any disagreement among researchers was resolved jointly and reviewed by a fourth researcher (MMR).

The Kappa coefficient proposed by Landis and Koch [28] was used to assess the agreement between researchers in the selection stages within a range from <0 to 1 in the following categories: <0 = no agreement; 0–0.20 = poor agreement; 0.21–0.40 = fair agreement; 0.41–0.60 = moderate agreement; 0.61–0.80 = substantial agreement; and 0.81–1.00 = almost perfect agreement.

Information extracted from the studies included: country, design of study, participants’ characteristics, identification of the microRNA, and quantification method; inflammatory, lipid and glycemic biomarkers, and anthropometric variables. Glycemic and lipid biomarkers were converted into mg/dL [12,21,29,30,31,32,33,34] and fasting insulin into µIU/mL.

Measures of central tendency and dispersion of circulating microRNA presented in graphs [9,12,13,16,21,30,31,33,34,35,36,37,38,39] were extracted using the Web Plot Digitizer software version 4.1 (Ankit Rohatgi, Austin, TX, USA). Indirect extraction methods [40,41] were applied to estimate mean and standard deviation if data were not informed in the studies [9,21,30,31,33,34,35,36,37].

Connections between microRNA and diseases identified in studies included in the systematic review were used to develop a complex network (graph) synthesizing the evidence obtained in the analysis. The complex network encompassed nodes of origin representing the microRNA investigated in the studies, nodes of destination representing the diseases studied, and connections between nodes (edges) representing studies that showed an association between microRNA expression and the diseases studied.

The sizes of nodes were assigned proportionally to the connections established (average degree) and the strength of connections was represented by the number of studies linking the microRNA expression and the diseases evaluated. The network was designed using the Fruchterman Reingold layout, which comprises a direct force algorithm representing nodes connected with higher intensity by proximity and presenting uniform distribution of network nodes to minimize intersections between arcs [42].

### 2.4. Quality Assessment of Studies

The quality of studies included in the systematic review was independently assessed by two researchers (PNBL and GBC) using the Quality Assessment Tool for Observational Cohort and Cross-Sectional Studies from the National Institutes of Health [43], which is based on 14 criteria for assessment of the study quality. Studies were categorized as good (≥12), fair (5–11), or poor (<5) quality [43]. Any disagreements were resolved through discussion between the researchers and reviewed by a third researcher (MMR).

## 3. Results

### 3.1. Characteristics of Studies

**Figure 1:**
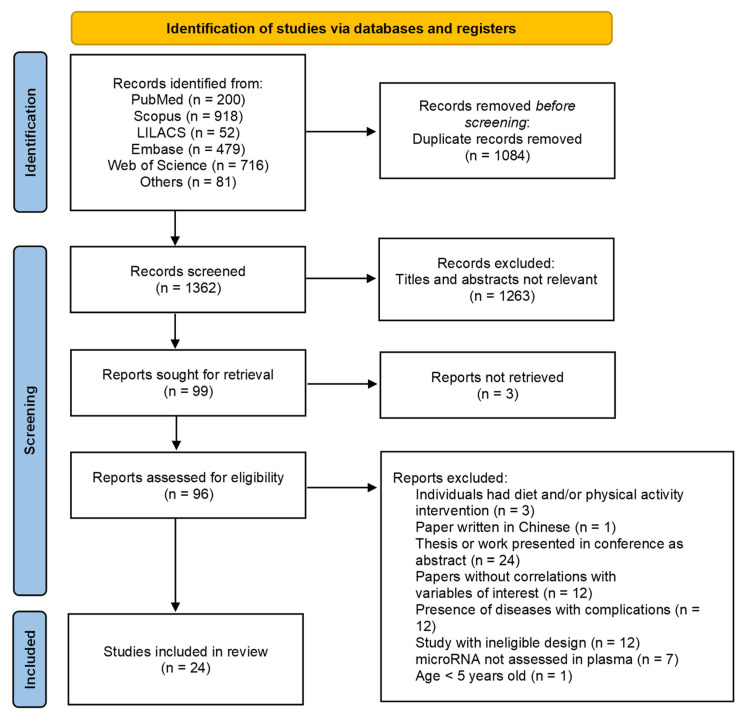
Flowchart of search and selection of studies in the systematic review.

The initial search identified 2446 studies, with 24 studies included in the systematic review [10,12,13,14,16,17,18,19,20,21,22,25,29,30,31,32,33,34,35,36,37,38,39]. Kappa coefficient indicated substantial agreement between researchers (0.669) in the first stage (title and abstract analysis) and almost perfect agreement (0.804) in the last stage (full-text analysis). The flowchart with details of study selection steps is presented in Figure 1.

The majority of studies (10 studies) was conducted in Asia: China [12,29,31,33], Taiwan [9], Japan [32], India [17,38], and Iran [16,39]. In addition, six studies were carried out in the Americas: USA [18,21,30,36], Mexico [35], and Chile [19]; and five studies were performed in Europe: Spain [10,25], Romania [34], Italy [37], and one multicentric study in Belgium, Cyprus, Estonia, Germany, Hungary, Italy, Spain, and Sweden [14]. Finally, three studies were conducted in Africa (Egypt) [13,20,22].

Overall, 1656 individuals were in the case group and 1152 individuals were in the control group. Two studies included children ≤10 years old [10,12], three studies included preadolescents/adolescents [13,14,19], and the remaining 19 studies included adults and older adults (Table 1).

Individuals were overweight or obese in 20 studies. Most studies reported glycemic, lipid, and anthropometric markers. Two studies did not include anthropometric variables [29,34], one study did not present body mass index (BMI) values [21], and one study did not present glycemic markers [22]. Six studies assessed inflammatory markers [10,13,16,34,35,36,39], mainly C-reactive protein (CRP) levels.

MicroRNAs were quantified in plasma (n = 12) or serum (n = 12) by RT-PCR and used different methods to control expression and normalize results (Table 2). Four studies informed the methodology used to control hemolysis [9,14,37,38].

**Figure 2:**
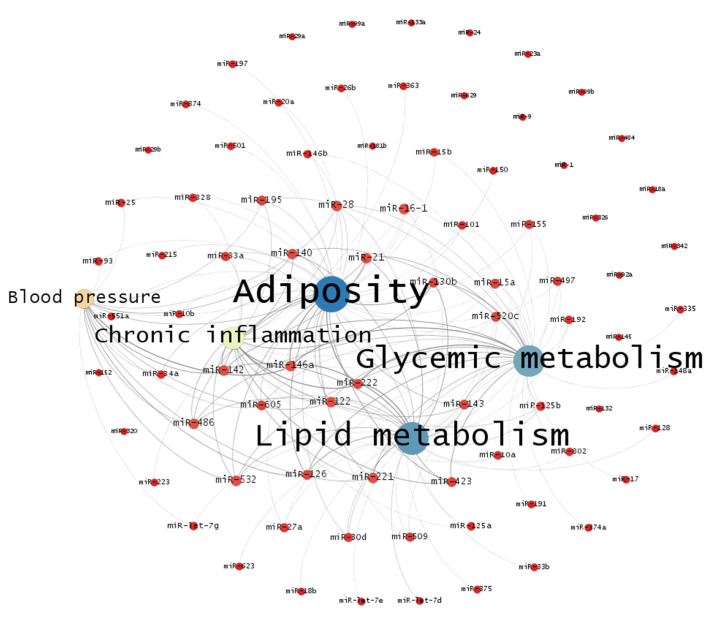
Network of connections between microRNA identified in studies included in the systematic literature review in relation to adiposity, chronic inflammation, blood pressure, and biomarkers related to lipid and glycemic metabolism.

Regarding the diseases of interest, 86 microRNAs were investigated in T2D, obesity, dyslipidemia, hypertension, and MetS. The disease most frequently investigated in the studies was T2D, including analysis of associations with 51 microRNAs, followed by obesity (35 microRNAs) and MetS with its risk factors (20 microRNAs). Considering the studies selected in the systematic review, a major part of the studies investigated the following microRNAs: miR-146a, miR-222, miR-126, miR-130b, miR-142, miR-423, miR-21, miR-532, miR-28, miR-122, miR-140, miR-143, and miR-486 (Figure 2), being related to three or more diseases.

The network of studies included in the systematic review showed that there was a higher number of studies linking microRNA with adiposity, lipid metabolism, and glycemic metabolism (Figure 2). Based on higher average weighted degree, robust connections between miR-146a and miR-122 were identified in relation to the conditions studied and miR-222 in relation to adiposity, lipid metabolism, glycemic metabolism, and low-grade chronic inflammation. Adiposity, lipid metabolism, and glycemic metabolism were also intensely linked to miR-126, miR-221, and miR-423. Numerous microRNAs were unconnected to health conditions investigated in the systematic review (Figure 2).

The average degree (1.791) and average weighted degree (3.988) of the graph showed that major part of the connections between nodes are still sparse and the low modularity (0.109) indicated the absence of robust structure in the network, i.e., there is a lack of groups of studies focusing on similar relationships between microRNA and the biomarkers evaluated in the systematic review. Thus, the absence of sufficient studies pairing similar microRNAs and biomarkers comprised an obstacle to perform a meta-analysis.

Specific aspects regarding microRNA expression according to risk factors and age groups are presented in subsequent sections (Table 2). Table 3 shows correlations between microRNA most frequently investigated in the selected studies and markers related to obesity, T2D, MetS, hypertension, and dyslipidemia.

### 3.2. Circulating microRNA Expression in Children with Obesity

Two studies [10,12] evaluated 475 children with normal weight (NW), overweight, and obesity (Table 1). Twenty-one microRNAs were assessed in serum and plasma, with fifteen upregulated and five downregulated in children with obesity (Table 2). Two of them, miR-486 and miR-222, were consistently upregulated in children with obesity in both studies included. It is important to notice that participants did not present chronic or acute illnesses and, therefore, did not use medication. Although the study of Prats-Puig et al. [10] included children with higher mean age, children in the study were not under pubertal development and the statistical analysis was adjusted by age.

Both studies showed correlations of miR-222 and miR-486 with BMI and central body adiposity [10,12] (Table 3). In addition, miR-222 and miR-486 showed correlations with HOMA-IR, adiponectin, and CRP [10] (Table 3). The detailed synthesis of the correlations is presented in Table S3.

### 3.3. Circulating microRNA Expression in Preadolescents and Adolescents with Obesity and Metabolic Syndrome

Three studies included evaluated plasma microRNA expression in 597 preadolescents and adolescents [13,14,19] (Table 1). The absence of other diseases associated with obesity was confirmed in only one of the studies [13]. The pubertal development stage was not mentioned in the studies.

Eight microRNAs were upregulated and nine downregulated in preadolescents/adolescents with obesity and/or MetS, with no overlap between studies [4,13,19] (Table 2). Three microRNAs (miR-126, miR-132, and miR-145) showed no differences in preadolescents/adolescents with MetS when compared with the control group without metabolic syndrome [19].

Seven microRNAs evaluated in the studies with children were replicated in preadolescents/adolescents, showing agreement in the upregulated expression of four (miR-142, miR-140, miR-222, and miR-130b). In contrast, three microRNAs (miR-532, miR-423, and miR-146a) showed downregulation in preadolescents/adolescents with obesity [13], contrarily to that reported in children with obesity [10,12].

Some upregulated microRNAs (miR-222, miR-140, miR-130) were positively associated with fasting blood glucose (FBG), insulin, and HOMA-IR values [13], while downregulated microRNAs (miR-146a, miR-532, miR-15a) were negatively associated with these variables [13] (Table 3). Positive correlations of microRNA expression were observed in relation to HDL-c, triglycerides (TG), and LDL-c levels [13].

Among the most frequently evaluated microRNAs (miR-222, miR-532, miR-146a, miR-130, miR-140), authors showed correlations with BMI [12,13], adiponectin [13], and leptin [13] levels (Table 3 and Table S3). There were high levels of miR-let-7e in preadolescents with MetS, showing values twice higher in those with higher insulinemia and HOMA-IR values. The increased levels of this microRNA were correlated with a progressive increase in the number of risk factors for MetS and reduced HDL-c levels [19]. Likewise, miR-126 was positively correlated with waist circumference, BMI, and TG [19] (Table 3 and Table S3).

### 3.4. Circulating microRNA Expression in Adults with Obesity without Metabolic Diseases Associated

The evidence showed increased levels of miR-122 and miR-34a in obese compared to normal-weight adults, whilst three microRNAs (miR-126, miR-146a and miR-150) had reduced levels (Table 2) [24,30,31]. Furthermore, expression of miR-181b showed no differences in different groups of individuals [30]. The expression of miR-122 in adults showed similar patterns in relation to results reported among obese children and the opposite was observed in relation to miR-146a.

Regarding covariates, obese adults included in the studies did not present chronic or acute illness or major abnormalities. In addition, women were not menopausal, pregnant, or breastfeeding [20,31]. Age and gender were considered in the analysis presented by one study [31]. Hijmans et al. 2018b did not include smokers and participants using medication [30].

A positive relation was observed between miR-34a and BMI, whereas miR-126, miR-146a, and miR-150 were inversely related to BMI [30]. Circulating miR-122 was positively correlated with BMI, body fat (%), blood pressure, and TG levels [31] and negatively correlated with HDL-c levels [24]. On the other hand, there were positive correlations of miR-122 [31] with FBG, insulin, and HOMA-IR values (Table 3).

### 3.5. Circulating microRNA in Metabolic Syndrome and Associated Factors

MetS is linked to increased central body fat, high blood pressure, insulin resistance, and changes in the lipid profile. In this context, there were increased expressions of miR-10a [34], miR-21 [34], miR-33a [34,35], miR-33b [35], miR-125a [34], and miR-146a [34] in individuals with insulin resistance and/or hyperlipidemia (Table 2). In the insulin-resistance condition, there were positive correlations of miR-33a with visceral fat, total cholesterol (TC), and TG levels [35]. Additionally, serum miR-10a, miR-21, miR-33a, miR-125a, and miR-146a levels were positively correlated with TC, TG, LDL-c, CRP, and IL-1β levels in individuals with hyperglycemia and hyperlipidemia [34] (Table 3 and Table S3).

Individuals with low HDL-c levels had decreased miR-221 levels and increased miR-222 levels compared to normolipidemic individuals [29] (Table 2). Furthermore, miR-221 was positively correlated with TC levels [19] (Table 3 and Table S3).

Individuals with hypertension presented reduced levels of miR-21, miR-126, and miR-146a, whilst miR-34a was increased in hypertensive individuals [21] (Table 2). Three of the four microRNAs evaluated (miR-21, miR-126, and miR-146a) showed negative correlations with blood pressure values and only miR-34a showed positive correlation with systolic blood pressure [21]. There were no correlations between the expression of these microRNAs and other cardiometabolic biomarkers [21] (Table 3 and Table S3).

Overweight/obese adults with hypertension showed increased levels of miR-605 and miR-623 [22]. There were positive correlations between TC and LDL-c levels (miR-605) and negative correlation with HDL-c levels (miR-623) [22] (Table S3).

Individuals with MetS had increased levels of miR-let-7g and miR-221 compared to individuals without MetS, expression that increased according to the presence of additional risk factors for MetS [9] (Table 2). The authors also indicated that the difference in microRNA levels was greater in women [9]. The miR-let-7g was inversely related to HDL-c levels and blood pressure values [9] (Table S3).

Elevated serum levels of miR-486, miR-497, miR-509, and miR-605 were observed in adult men with metabolic syndrome [20] (Table 2). In this condition, miR-486, miR-497, and miR-509 were positively correlated with waist circumference, FBG, and TG levels, in addition to negative correlation with blood pressure values [20]. Conversely, miR-605 was negatively correlated with waist circumference and TG levels and positively correlated with blood pressure values [20] (Table S3).

### 3.6. Circulating microRNA in Adults and Older Adults with Type 2 Diabetes

Twenty microRNAs showed increased levels and seventeen microRNAs showed reduced levels in individuals with T2D. Both positive and negative regulation were observed for miR-21, miR-24, miR-27a, miR-30d, miR-130b, and miR-222 (Table 2). It is important to emphasize that individuals with T2D were overweight and/or obese in the studies evaluated.

Negative correlations between levels of miR-21, miR-27a, miR-30d, and miR-155 were observed in relation to total and central body adiposity [16,37,39] (Table 3 and Table S3). Only miR-101 was positively correlated with BMI in Japanese individuals with T2D [32] (Table S3).

Direct and inverse correlations between microRNA levels and glycemic variables were observed in individuals with T2D and overweight/obesity (Table 3 and Table S3). FBG, insulin, HbA1c, and HOMA-IR values showed positive correlations with miR-140 and miR-142 [25,37] and were negatively associated with miR-21 [16,39], miR-126 [25], miR-146a [33], miR-423, and miR-532 [25] (Table 3).

It should be noted that positive and negative correlations were observed for miR-130b and miR-222 in the studies evaluated (Table 3 and Table S3). There was a positive correlation between miR-130b and HbA1c in normoglycemic and newly diagnosed T2D adults without the use of medication [38]; however, miR-130b was negatively correlated with HbA1c in adults and older-adult men with T2D [25].

Adults and older adults with newly diagnosed T2D showed negative correlation between miR-222 and HbA1c [37], whilst adult and older-adult men with established T2D had positive correlations between miR-222 and HbA1c and FBG [25].

Increased levels of miR-130b, miR-423, and miR-532 were related to lower TG levels [25]. In contrast, increased levels of miR-140 and miR-142 were positively related to the increase in TG levels [25]. Furthermore, miR-423 was negatively correlated with HDL-c levels [38] and miR-21 showed negative correlation with total cholesterol (TC) and positive correlation with HDL-c [16,39]. On the other hand, miR-28 was positively correlated with TC and negatively correlated with LDL-c in individuals with newly diagnosed T2D without the use of medication [37] (Table 3 and Table S3).

Only one study investigated the relationship between microRNA levels and inflammatory biomarkers in individuals with T2D [36]. The authors showed that miR-24 and miR-27a presented, respectively, negative and positive correlations with IL-8 levels. Furthermore, there was positive correlation between miR-34a and IL-6 level and positive correlations between miR-29b and miR-155 in relation to IL-12 levels (Table S3).

Time of T2D diagnosis may influence microRNA levels [25,37] and three studies evaluated individuals newly diagnosed with T2D who were not using antidiabetic drugs [33,37,38]. Nevertheless, some studies selected individuals with established T2D, continuous use of medication, and the use of insulin was not an exclusion factor for subjects [16,25,32,36,39]. Only one study considered that excessive alcohol consumption (>3 drinks/day) and smoking should be exclusion criteria [36].

### 3.7. Assessment of the Quality of Studies in the Systematic Review

In this systematic review, 24 studies presented 5 to 11 points, being categorized as fair quality [43]. The main criteria impacting the results of quality assessment were incomplete data on the population of study and the lack of sample size justification. In addition, the quality assessment tool is applicable in cross-sectional and cohort studies; therefore, some questions were not applicable to the studies included in the systematic review.

## 4. Discussion

The systematic review synthesized the evidence on circulating microRNAs related to risk factors for MetS in individuals ≥5 years old. Eleven of the thirteen microRNAs most frequently investigated were associated with lipid, anthropometric, glycemic, and inflammatory variables in individuals in different life stages. Overweight/obesity was often observed in the studies included in the systematic review, which suggests that metabolic alterations caused by the total (BMI) and central (waist circumference) adiposity may be responsible for the change in circulating microRNA levels [10,12,13,14,31].

The miR-130b, a potential biomarker for obesity, was related to lipid, glycemic, and inflammatory metabolism, suggesting that miR-130b may be associated with impaired metabolic control [9]. The miR-130b is secreted by adipose tissue and mediates the metabolic regulatory action of TGF-β, which acts on body energy homeostasis [9,44]. Other mechanisms related to body-weight gain are the JAK-STAT and MAPK pathways, in which the action of miR-140 is observed [45]. There was no evidence of significant associations of miR-21 with body adiposity in humans; however, in vitro studies showed that miR-21 was involved both in TGF-β pathway and adipocyte differentiation [46,47,48].

Likewise, some microRNAs are linked to obesity-induced inflammation through pro-inflammatory pathways, such as NF-κB and the pro-inflammatory cytokines TNFα and IL-6 [10,49,50,51]. Four of the studies identified relationships between the expression of circulating microRNAs (e.g., miR-21, miR-122, miR-130b, miR-142, miR-146a miR-486, and miR-523) and the inflammatory biomarkers CRP [10,34], IL-1β [34], IL-6 [36], adiponectin [10,13], and leptin [13]. In vitro studies with animal and human cells showed that the expression of miR-146a, miR-486, and miR-532 was regulated in response to the NF-κB signaling pathway. In addition, hyperglycemia and insulin resistance may alter levels of miR-146a and miR-486 [10,49,50,51].

Indeed, miR-122 showed robust connections with risk factors for MetS investigated in the systematic review, in accordance with predicted target genes, highlighting its participation in lipid oxidation and hepatic synthesis of fatty acid and cholesterol [23,52]. In addition, considering the correlation with adiponectin levels, which regulate the production of TNFα and IL-6 [53], it may play an important role in inflammatory processes. Similarly, miR-126 had altered expression in obesity and may modulate CCL2 (chemokine ligand 2) through genes that encode ETS1, MAX, NFKB1, RELB, and STAT6 proteins [54,55,56,57,58]. The miR-126 has been consistently associated with T2D in the literature [59] and has been shown to regulate vascular integrity and angiogenesis [59] through Notch1 inhibitor delta-like 1 homolog (Dlk1) [60] and the argonaute-2 (Ago2)/Mex3a complex [61]. The interaction of miR-486 and miR-142 in the forkhead box O1 transcription factor inhibition was also identified [10] and participation with other microRNAs in the phosphatase and tensin homolog protein (PTEN) pathway and consequent activation of the PI3K/Akt [62,63].

The miR-146a showed increased levels in obese children [12], conversely to individuals with overweight/obesity in different age groups [13,30], thus, demonstrating that age may be an important factor in the evaluation of circulating microRNAs. Although the direct relationship between miR-146a and aging has not been demonstrated in the studies analyzed in the systematic review, some microRNAs may regulate cellular senescence at the post-transcriptional level. For example, in human mammary epithelial cells, miR-130b repressed p21 expression [64]. In addition, a previous study identified microRNAs (miR-142-5p, miR-222) related to the aging process through different cellular damage pathways in human serum samples [65].

Different patterns of miR-130b and miR-222 were observed in individuals with obesity, isolated or associated with T2D [25,37,38]. Increased levels of miR-130b were associated with long-term glycemic alterations (HbA1c) in adults with obesity and newly diagnosed T2D [38]. On the other hand, miR-130b levels showed negative correlations with glycemic biomarkers in adults and older adults with obesity and established T2D [25]. Similarly, miR-222 was positively correlated with elevated glycemic biomarkers in adults or older adults with T2D and inversely correlated in newly diagnosed individuals [37].

A potential explanation for the differences observed in the studies may be the initial compensatory mechanisms that precede pancreatic failure, marked by increases in insulin synthesis and release by pancreatic beta cells to re-establish glycemic homeostasis [45]. Thus, microRNA involved in beta cell mass control, insulin secretion, and signaling mechanisms respond to the glycemic imbalance conditions [66,67,68].

The expression levels of miR-221 were increased in individuals with MetS, being proportional to the number of risk factors for MetS [9]. Based on predicted target genes, miR-221 was related to inflammatory response, cell signaling, and insulin metabolism, presenting complementary action in relation to miR-222, since they are homologous microRNAs [29,52].

Challenges remain for the use of circulating microRNAs as biomarkers for MetS, considering that a single microRNA may be regulated by multiple factors. An important aspect to be discussed is the potential influence of medical treatments on the results (Table 1). The use of antidiabetic agents by individuals with T2D may influence the expression of circulating microRNA [25,69,70,71]. Results of one study included in the systematic review showed that metformin altered the plasma expression of miR-140, miR-142, and miR-222 in individuals with T2D [25]. Additionally, antihypertensives, another class of drugs largely utilized by individuals with MetS, have been associated with microRNA expression in previous studies [72,73].

Furthermore, sex may influence gene expression and microRNA regulation under different physiological conditions, due to genes linked to the X chromosome and action of sexual hormones [74,75,76]. Potential functional variants in the genome have been identified that may justify differential gene expression between sexes [76] and sexual dimorphism observed in some diseases [74]. It has been suggested that sex steroid hormones (e.g., estrogen) may regulate ribonucleases Drosha and Dicer and the expression of argonaut proteins, thus, indicating their role in post-transcriptional processing of microRNA [75]. However, there was an absence of evidence on differences in circulating microRNA levels due to sex in the systematic review [13,14,16,22,29,31,33,36,39].

Although a considerable number of microRNAs were assessed in studies included in the systematic review, studies were marked by high heterogeneity, with few studies identified that evaluated similar circulating microRNAs in association with the same clinical conditions.

The studies included in the systematic review adopted several strategies for normalization of microRNA expression, ranging from the use of synthetic spike-in or identification of relatively stable endogenous circulating oligonucleotides to applying an average of cycle thresholds. Some inconsistencies identified in studies screened in the systematic review might be explained by the absence of a standardized normalization method [77].

Moreover, nutritional aspects may influence the expression of circulating microRNAs, in addition to clinical and lifestyle characteristics considered in the systematic review [77] and, thus, should be considered in future studies due to their role as modifiable risk factors for the development of NCD.

## 5. Conclusions

Circulating microRNAs were mainly related to adiposity, lipid metabolism, and glycemic metabolism, showing distinct expression profiles according to the clinical condition of individuals. We highlighted the connections between miR-122, miR-126, miR-146a, miR-221, miR-222, and miR-423 expressions and risk factors for MetS. In addition, excess body fat was often observed in studies included in the systematic review, potentially playing a key role in circulating microRNA dysregulation.

Although there were numerous studies identified in the literature, the high heterogeneity of studies investigating the association between microRNA and MetS risk factors prevented further exploration of factors responsible for variations in microRNA expression. Therefore, further studies are required to allow for the identification of potential associations between circulating microRNAs and risk factors for MetS.

## Figures and Tables

**Table 1:** Characterization of studies included in the systematic review.

Author/Year	Country	Presence of Diseases	Pharmacological Treatment	Total Sample (M/F)	Case Group (M/F)	Control Group (M/F)	Age (Years)	BMI (kg/m^2^)	General Factors	Analysis Adjustment
Children										
Cui et al., 2018 ^&^ [12]	China	Obesity	Non-medicated	172/180	98/108	74/72	5.0 ± 0.9	Overweight:17.4 ± 0.6 Obese: 20.3 ± 2.2 Healthy controls: 15.1 ± 1.06	Without chronic or acute illness or major abnormalities	No
Prats-Puig et al., 2013 [10]	Spain	Obesity	Non-medicated	61/64	18/22	43/42	9.5 ± 1.3	Obese: 3.36 ± 0.43 Lean: −0.62 ± 0.30	No pubertal development, stable weight for height, without chronic or acute illness or major abnormalities	Age
Preadolescents and adolescents										
Krause et al., 2015 [19]	Chile	Metabolic Syndrome	Not informed	66/92 *	128 *	30 *	11.6 ± 0.9	All sample: 24.6 ± 4.0	Control group without metabolic syndrome traits	No
Iacomino et al., 2019 [14]	Eight European countries ^#^	Obesity	Not informed	86/103	41/53	45/50	12.2 ± 1.7	Overweight/obese: 1.75 ± 0.61 NW: −0.04 ± 0.50	Unclear on the presence of diseases and use of medications	Gender, age and country
Al-Rawaf, 2018 [13]	Egypt	Obesity	Non-medicated	150/100	122/78	28/22	13.9 ± 2.9	Overweight: 21.9 ± 5.7 Obese: 26.7 ± 8.2 NW: 17.4 ± 4.3	Without chronic or acute illness or major abnormalities	Age
Adults and older adults										
Wang et al., 2015 [31]	China	Obesity	Not informed	112/118	62/61	50/57	24.0 ± 2.7	Obese: 37.73 ± 4.40 NW: 20.79 ± 1.41	Without chronic or acute illness or major abnormalities	Age, gender, HDL-c and alanine aminotransferase
Hijmans et al., 2018 [30]	USA	Obesity	Non-medicated	23/22	15/15	8/7	55.0 ± 1.4	Normal weight: 23.3 ± 1.2 Overweight: 28.2 ± 1.2 Obese: 32.3 ± 1.9	Sedentary, non-hypertensive, non-smokers, normolipidemic, without chronic or acute illness or major abnormalities	No
Wang et al., 2013 [9]	Taiwan	Metabolic syndrome	Non-medicated for hyperglycemia or hyperlipidemia	52/50	16/15	36/35	55.8 ± 8.0	With MetS: 26.6 ± 3.6 Without MetS: 24.1 ± 3.3	Without chronic or acute illness or major abnormalities	Age, gender and smoking
Zaki et al., 2019 [20]	Egypt	Metabolic Syndrome	Not informed	75/0	55/0	20/0	18–50	Not informed	Without chronic or acute illness or major abnormalities	No
Zhou et al., 2018 [29]	China	↓ HDL-c	Non-medicated	90/84	45/43	45/41	<55	Not informed	Without obesity, metabolic syndrome, chronic or acute illness or major abnormalities	Gender
Simionescu et al., 2014 [34]	Romania	Dyslipidemia Hyperglycemia	Not informed	10/15	8/12	2/3	56.2 ± 12.3	Not informed	Groups: hyperlipidemic, hyperglycemic, hyperlipidemic/hyperglycemic and control (normolipidemic/ normoglycemic)	No
Badawy; Abo-Elmatty; Mesbah, 2018 [22]	Egypt	Hypertension	Anti-hypertensive medication	24/26	13/12	11/14	50.2 ± 11.0	Hypertensive: 26.7 ± 3.7 Normotensive: 27.5 ± 4.13	Included smoking	No
Hijmans et al., 2018 [21]	USA	Hypertension	Non-medicated	20/10	10/5	10/5	57.5 ± 1.6	Hypertensive: 25.8 ± 2.7 Normotensive: 25.2 ± 2.7	Sedentary, non-hypertensive, non-smokers, normolipidemic, without chronic or acute illness or major abnormalities	No
Rong et al., 2013 [33]	China	T2D	Non-medicated	94/86	47/43	47/43	48.3 ± 10.3	New-T2D: 24.58 ± 3.66 NGT: 23.38 ± 2.95	BMI < 40 and without chronic or acute illness or major abnormalities	Age, gender, BMI, smoking, alcohol drinking, history of hypertension, family history of diabetes, and specific biochemical indicators
Ortega et al., 2014 [25]	Spain	T2D Obesity	Not informed	93/0	58/0	35/0	52.1 ± 10.2	NGT/NW: 25.2 ± 1.8 T2D/NW: 26.4 ± 2.4 NGT/obese: 32.2 ± 2.4 T2D/obese: 33.4 ± 3.3	Stable metabolic control	Age, BMI
Higuchi et al., 2015 [32]	Japan	T2D	Metformin, insulin, α-glucosidase inhibitors, sulfonylureas, pioglitazone, glinides and DPP-4 inhibitors	121/83	96/59	25/24	58.4 ± 14.2	T2D: 25.9 ± 4.97 NGT: 23.6 ± 4.05	Without renal dysfunctions	Age, HbA1c, postprandial glucose, BMI, TG, HDL-c and glomerular filtration rate
Prabu et al., 2015 [38]	India	T2D	Non-medicated	74/71	48/48	26/23	44.3 ± 7.4	IGT: 24.9 ± 2.9 T2D: 25.7 ± 3.5 NGT: 24.5 ± 2.6	Newly diagnosed T2D	Gender
Lopez; Garufi; Seyhan, 2016 [36]	USA	Obesity T2D	Only one of them: metformin, sulfonylureas, Glucagon-like peptide-1 analogs and/or Dipeptidyl peptidase IV inhibitors	26/32	21/17	5/15	45.2 ± 29.5	Control/lean: 22.5 ± 3.7 Pre-T2D/lean: 21.6 ± 4.5 T2D/lean: 23.1 ± 0.6 Control/obese: 31.9 ± 8.3 Pre-T2D/obese: T2D/obese: 41.5 ± 21.1	Without chronic or acute illness, major abnormalities or drugs/alcohol use	BMI, age and gender
Candia et al., 2017 [37]	Italy	T2D	Non-medicated	11/16	7/11	4/5	60.3 ± 8.1	NGT: 23.7 ± 3.3 IGT: 25.6 ± 3.3 T2D: 29.6 ± 7.8	Newly diagnosed T2D	No
Ghorbani et al., 2018 [39] and Mahdavi et al., 2018 ^€^ [16]	Iran	T2D Obesity	Metformin, statins, antihypertensive	39/50	26/21	13/29	52.2 ± 7.1	T2D: 28.2 ± 4.8 NGT: 27.3 ± 3.9	Without chronic or acute illness or major abnormalities	BMI, age and gender
Sucharita et al., 2018 [17]	India	T2D	Oral hypoglycemic agents	42/18	21/9	21/9	46.3 ± 7.1	T2D: 27.3 ± 4.6 NGT: 27.3 ± 4.7	Duration of disease < 5 years, without chronic or acute illness or major abnormalities	Age
Williams et al., 2019 [18]	USA	Obesity T2D	Not informed	0/67	0/44	0/23	61.3 ± 1.1	NGT: 30.5 ± 6.2 T2D: 38.1 ± 8.4	Insufficient information	No
Corona-Meraz et al., 2019 [35]	Mexico	Insulin resistance	Non-medicated	24/56	25 **	55 **	20–59	Non-IR young: 26.2 ± 5.7 Non-IR senior: 28.0 ± 4.5 IR young: 33.9 ± 7.1 IR senior: 31.1 ± 6.5	Without chronic or acute illness or major abnormalities	No

Age is presented as mean ± standard deviation or range. BMI Z-score: references [10,14]. NGT, Normal glucose tolerance; NW, Normal weight; T2D, type 2 diabetes; IR, Insulin resistance; HDL, high-density lipoprotein. * The study showed contradictory information regarding sample size, and did not report the number of M/F per group. ** The study did not report the number of M/F per group. ^&^ Cross-sectional arm. ^€^ Papers with the same sample. ^#^ Belgium, Cyprus, Estonia, Germany, Hungary, Italy, Spain, and Sweden.

**Table 2:** Characteristics of microRNA evaluated in the papers included in the systematic review, according to disease.

Author/Year	Disease	Sample	Normalization	Regulation
Children				
Cui et al., 2018 ^&^ [12]	Obesity	Serum	2^−ΔΔCt^ method, using syn-cel-miR-39 as reference	↑ miR-222, miR-486, miR-146b, miR-146a, miR-20a, miR-15b, miR-26b ↓ miR-197
Prats-Puig et al., 2013 [10]	Obesity	Plasma	ΔCt method, using miR-106a, miR-146a, miR-19b, and miR-223 as reference	↑ miR-486-5p, miR-486-3p, miR-142-3p, miR-130b, miR-423-5p, miR-532-5p, miR-140-5p, miR-16-1, miR-222, miR-363, miR-122 ↓ miR-221, miR-28-3p, miR-125b, miR-328 ↔ miR-195
Preadolescents and adolescents				
Krause et al., 2015 [19]	Metabolic syndrome	Plasma	2^−ΔΔCt^ method, using syn-cel-miR-39 as reference	↑ miR-let-7e ↔ miR-126, miR-132, miR-145
Al-Rawaf, 2018 [13]	Obesity	Plasma	2^−ΔCt^ method, using cel-RNU43 as endogenous reference	↑ miR-142-3p, miR-140-5p, miR-222, miR-143, miR-130b ↓ miR-532-5p, miR-423-5p, miR-520c-3p, miR-146a, miR-15a
Iacomino et al., 2019 [14]	Obesity	Plasma	Geometric mean, using spike-in-Cel-miR-39 and SNORD95 as reference	↑ miR-501-5p, miR-551a ↓ miR-10b-5p, miR-191-3p, miR-215-5p, miR-874-3p
Adults and older adults				
Wang et al., 2015 [31]	Obesity	Serum	Quantile algorithm (Gene Spring Software 11.0—Agent Technologies), using SYBR green as reference	↑ miR-122
Hijmans et al., 2018b [30]	Obesity	Plasma	ΔCt method, using cel-miR-39 as reference	↓ miR-126, miR-146a, miR-150 ↑ miR-34a ↔ miR-181b
Wang et al., 2013 [9]	Metabolic syndrome	Serum	Median normalization method, using syn-cel-lin-4 as reference	↑ miR-let-7g, miR-221
Zaki et al., 2019 [20]	Metabolic Syndrome	Serum	2^−ΔΔCt^ method, using SNORD68 as reference	↑ miR-486-5p, miR-497, miR-509-5p, miR-605
Simionescu et al., 2014 [34]	Dyslipidemia Hyperglycemia	Serum	2^−ΔCt^ method, using cel-miR-39 as reference	↑ miR-125a-5p, miR-146a, miR-10a, miR-21, miR-33a
Zhou et al., 2018 [29]	↓ HDL-c	Plasma	2^−ΔCt^ method, using miR-191-5p as reference	↑ miR-222-3p ↓ miR-221-3p
Badawy;Abo-Elmatty; Mesbah, 2018 [22]	Hypertension	Serum	2^−ΔΔCt^ method, using miR U6 as reference	↑ miR-605, miR-623
Hijmans et al., 2018 [21]	Hypertension	Plasma	ΔCt method, using cel-miR-39 as reference	↓ miR-21, miR-126, miR-146a ↑ miR-34a ↔ miR-17, miR-92a, miR-145, miR-150
Rong et al., 2013 [33]	T2D	Plasma	2^−ΔΔCt^ method, using miR-16 as reference	↑ miR-146a
Ortega et al., 2014 [25]	T2D Obesity	Plasma	Geometric mean method, using miR-106a, miR-146a, miR-19b, and miR-223 as reference	↑ miR-140-5p, miR-142-3p, miR-222 ↓ miR-423-5p, miR-125b, miR-192, miR-195, miR-130b, miR-532-5p, miR-126
Higuchi et al., 2015 [32]	T2D	Serum	Log 10 transformation, using C. elegans spiked-in control miRNA and cel-miR-39 as reference	↑ miR-101, miR-375, miR-802 ↔ miR-335
Prabu et al., 2015 [38]	T2D	Serum	2^−ΔCt^ method, using RNA spike-in control (Sp6) as reference	↑ miR-128, miR-130b-3p, miR-374a-5p, miR-99b ↓ miR-423-5p ↔ miR-629a-5p, let-7d-3p, miR-142-3p, miR-484
Lopez; Garufi; Seyhan, 2016 [36]	ObesityT2D	Plasma	−ΔΔCt method, using cel-miR39, miR-191, miR-423-3p, and miR-451 as reference	↑ miR-21, miR-24.1, miR-27a, miR-34a, miR-146a, miR-148a, miR-223, miR-326, miR-152 ↓ miR-29b, miR-126, miR-155, miR-25, miR-93, miR-150
Candia et al., 2017 [37]	T2D	Plasma	2^(average Ct-assay Ct)^ and log transformed, using UniSp2, UniSp4, UniSp5, and UniSp6 as reference	↑ miR-122, miR-148, miR-99 ↓ miR-18a, miR-18b, miR-23a, miR-24, miR-27a, miR-28, miR-30d, miR-222, miR-let-7d ↔ miR-126-3p
Ghorbani et al., 2018 [39] and Mahdavi et al., 2018 ^€^ [16]	T2D Obesity	Serum	2^−ΔCt^ method, using miR-39 and miR-16 as reference	↓ miR-21, miR-155 ↔ miR-126, miR-146a
Sucharita et al., 2018 [17]	T2D	Plasma	ΔCt method, using miR-16 as reference	↑ miR-30d ↔ miR-9, miR-1, miR-133a, miR-29a, miR-143
Corona-Meraz et al., 2019 [35]	Insulin resistance	Serum	2^−ΔCt^ method, using hsa-miR-320a as reference	↑ miR-33a, miR-33b
Williams et al., 2019 [18]	Obesity T2D	Serum	ΔCt method, using cel-hsa-miR-221-3p as reference	↓ miR-17

T2D, type 2 diabetes; HDL, high-density lipoprotein. ↑ indicates upregulation. ↓ indicates downregulation. ↔ No differences. ^&^ Cross-sectional arm. ^€^ Papers with the same sample.

**Table 3:** Correlations between metabolic biomarkers and main microRNAs evaluated in the papers included in the systematic review.

MicroRNA	Body Fluids	Characteristics of the Sample	Glycemic Variables	Lipid Variables	Inflammatory Variables	Anthropometric Variables	References
miR-21 *	Serum	T2D and obesity Adults and elderly	Insulin (−) HOMA-IR (−)	TC (−) HDL-c (+)	No correlations	BMI (−) WC (−)	[16,39]
	Serum	Dyslipidemia and hyperglycemia Young, adult and elderly	No correlations	TC (+) TG (+) LDL-c (+)	CRP (+) IL-1ꞵ (+)	Not evaluated	[34]
	Plasma	T2D and obesity Adults and elderly	Glucose (+) HbA1c (+)	No correlations	IL-6 (+)	No correlations	[36]
	Plasma	Hypertension Adults and elderly	No correlations	No correlations	Not evaluated	SBP (−)	[21]
miR-28	Plasma	Newly diagnosed T2D Adults and elderly	No correlations	TC (+) LDL-c (−)	Not evaluated	No correlations	[37]
	Plasma	Obesity Children	No correlations	No correlations	CRP (−) Adiponectin (+)	BMI (−) WC (−) BP (−)	[10]
miR-122 *	Plasma	Obesity Children	No correlations	No correlations	Adiponectin (−)	BMI (+) SBP (+)	[10]
	Serum	Obesity Young	FBG (+) Insulin (+) HOMA-IR (+)	TG (+) HDL-c (−)	Not evaluated	BMI (+) BP (+)	[31]
miR-126	Plasma	Obesity Adults and elderly	No correlations	No correlations	Not evaluated	BMI (−)	[30]
	Plasma	Hypertension Adults and elderly	No correlations	No correlations	Not evaluated	SBP (−)	[21]
	Plasma	Metabolic syndrome Children	No correlations	TG (+) VLDL-c (+)	No correlations	WC (+) BMI (+)	[19]
	Plasma	T2D and obesity Adults and elderly	FBG (−) HbA1c (−)	No correlations	Not evaluated	No correlations	[25]
miR-130b *	Plasma	T2D and obesity Adults and elderly	FBG (−) HbA1c (−)	TG (−)	Not evaluated	No correlations	[25]
	Plasma	Obesity Children	HOMA-IR (+)	HDL-c (−)	CRP (+)	BMI (+) WC (+)	[10]
	Serum	Newly diagnosed T2D Adults	HbA1c (+)	No correlations	Not evaluated	No correlations	[38]
	Plasma	Overweight and obesity Adolescents	FBG (+) Insulin (+) HOMA-IR (+)	TG (+) LDL-c (+) HDL-c (+)	Adiponectin (+) Leptin (+)	BMI (+)	[13]
miR-140	Plasma	Overweight and obesity Adolescents	FBG (+) Insulin (+) HOMA-IR (+)	TG (+) LDL-c (+) HDL-c (+)	Adiponectin (+) Leptin (+)	BMI (+)	[13]
	Plasma	T2D and obesity Adults and elderly	FBG (+) HbA1c (+)	TG (+)	Not evaluated	No correlations	[25]
	Plasma	Obesity Children	No correlations	No correlations	Adiponectin (−)	BMI (+) WC (+) BP (+)	[10]
miR-142	Plasma	Overweight and obesity Adolescents	FBG (+) Insulin (+) HOMA-IR (+)	TG (+) LDL-c (+) HDL-c (+)	Adiponectin (+) Leptin (+)	BMI (+)	[13]
	Plasma	T2D and obesity Adults and elderly	FBG (+) HbA1c (+)	TG (+)	Not evaluated	No correlations	[25]
	Plasma	Obesity Children	No correlations	No correlations	CRP (+) Adiponectin (−)	BMI (+) WC (+) BP (+)	[10]
miR-143	Plasma	Overweight and obesity Adolescents	FBG (+) Insulin (+) HOMA-IR (+)	TG (+) LDL-c (+) HDL-c (+)	Adiponectin (+) Leptin (+)	BMI (+)	[13]
miR-146a	Serum	Overweight and obesity Adults and elderly	No correlations	No correlations	Not evaluated	BMI (+)	[12]
	Plasma	Obesity Adults and elderly	No correlations	No correlations	Not evaluated	BMI (−)	[30]
	Plasma	Arterial hypertension Adults and elderly	No correlations	No correlations	Not evaluated	BP (+)	[21]
	Plasma	Overweight and obesity Adolescents	FBG (−) Insulin (−) HOMA-IR (−)	TG (+) HDL-c (+) LDL-c (+)	Adiponectin (+) Leptin (+)	BMI (−)	[13]
	Plasma	T2D and obesity Adults	HOMA-B (−)	No correlations	Not evaluated	No correlations	[33]
	Serum	Dyslipidemia and hyperglycemia Young, adult and elderly	No correlations	TG (+) TC (+) LDL-c (+)	CRP (+) IL-1ꞵ (+)	Not evaluated	[34]
miR-222 *	Plasma	Overweight and obesity Adolescents	FBG (+) Insulin (+) HOMA-IR (+)	TG (+) HDL-c (+) LDL-c (+)	Adiponectin (+) Leptin (+)	BMI (+)	[13]
	Serum	Overweight and obesity Adults and elderly	No correlations	No correlations	Not evaluated	BMI (+)	[12]
	Plasma	T2D and obesity Adults and elderly	FBG (+) HbA1c (+)	No correlations	Not evaluated	No correlations	[25]
	Plasma	Obesity Children	HOMA-IR (+)	TG (+) HDL-c (−)	CRP (+)	BMI (+) WC (+)	[10]
	Plasma	Newly diagnosed T2D Adults and elderly	HbA1c (−)	No correlations	Not evaluated	No correlations	[37]
	Plasma	Reduced HDL-c Adults	No correlations	HDL (−)	Not evaluated	No correlations	[29]
miR-423	Plasma	Overweight and obesity Adolescents	FBG (−) Insulin (−) HOMA-IR (−)	TG (+) LDL-c (+) HDL-c (+)	Adiponectin (+) Leptin (+)	BMI (−)	[13]
	Plasma	T2D and obesity Adults and elderly	FBG (−) HbA1c (−)	TG (−)	Not evaluated	No correlations	[25]
	Serum	Newly diagnosed T2D Adults	No correlations	HDL-c (−)	Not evaluated	No correlations	[38]
	Plasma	Obesity Children	HOMA-IR (+)	TG (+)	No correlations	BMI (+) WC (+)	[10]
miR-486 *	Serum	Overweight and obesity Adults and elderly	No correlations	No correlations	Not evaluated	BMI (+)	[12]
	Plasma	Obesity Children	HOMA-IR (+)	TG (+) HDL-c (−)	CRP (+) Adiponectin (−)	BMI (+) WC (+) BP (+)	[10]
	Serum	Metabolic syndrome Adults	FBG (+)	TG (+)	Not evaluated	BP (−)	[20]
miR-532	Plasma	Overweight and obesity Adolescents	FBG (−) Insulin (−) HOMA-IR (−)	TG (+) LDL-c (+) HDL-c (+)	Adiponectin (+) Leptin (+)	BMI (−)	[13]
	Plasma	T2D and obesity Adults and elderly	FBG (−) HbA1c (−)	TG (−)	Not evaluated	No correlations	[25]
	Plasma	Obesity Children	HOMA-IR (+)	TG (+)	CRP (+)	BMI (+) WC (+) BP (+)	[10]

* MicroRNA sequence -3p or -5p. FBG, fasting blood glucose. HOMA-IR, homeostasis model assessment of insulin resistance. HbA1c, glycated hemoglobin. TG, triacylglycerols. VLDL, very-low-density lipoprotein. HDL, high density lipoprotein. LDL, low density lipoprotein. TC, total cholesterol. T2D, type 2 diabetes. CRP, c-reactive protein. BMI, body mass index. SBP, systolic blood pressure. BP, Blood pressure. WC, waist circumference.

## Supplementary Materials

The following supporting information can be downloaded at: https://www.mdpi.com/article/10.3390/metabo12111044/s1, Table S1: Key terms used in database searches; Table S2: Papers excluded after full reading and reasons for exclusion; Table S3: Associations between microRNA and risk factors for metabolic syndrome.
