# Supplementary material for: Circulating microRNA Related to Cardiometabolic Risk Factors for Metabolic Syndrome: A Systematic Review
Source: Metabolites. 2022 Oct 30;12(11):1044. doi: 10.3390/metabo12111044 (PMC9692352; doi:10.3390/metabo12111044)
Supplement: Supplementary file 1 [file metabolites-12-01044-s001.zip › Table S1_Search Strategy_Supplementary data.pdf]

Supplementary data

Table S1 Key terms used in database searches.

Variable of interest	Key terms
**microRNA**	(“Circulating MicroRNA” OR “Circulating MicroRNAs” OR “MicroRNA, Circulating” OR " circulating miRNAs" OR “Blood microRNA” OR “Cell-Free MicroRNA” OR “Cell Free MicroRNA” OR “MicroRNA, Cell-Free" OR “hsa-miR” OR “serum MicroRNA” OR “plasma MicroRNA”)
**Lipid control variables**	(“Cholesterol, HDL” OR “HDL Cholesterol” OR “Cholesterol, LDL” OR “LDL Cholesterol” OR “lipoprotein cholesterol” OR “Cholesterol, VLDL” OR “VLDL Cholesterol” OR "lipid profile" OR triglycerides OR Triacylglycerol OR Triacylglycerols)
**Glycemic control variables**	(“blood glucose” OR glycemia OR fructosamine OR HbA1c OR “hemoglobin A1c” OR “Glycated Hemoglobin A” OR “glycosylated hemoglobin” OR insulin OR “homeostasis model assessment” OR HOMA)
**Inflammatory variables**	(“C-reactive protein” OR “C Reactive Protein” OR “Protein, C-Reactive" OR adiponectin OR Interleukin OR Interleukins OR “interleukin-6” OR “Interleukin 6 “ OR “IL6” OR “IL-6” OR “interleukin-10” OR IL-10 OR “IL 10” OR “Leptin” OR “Cell Adhesion Molecule-1” OR “Vascular Cell Adhesion Molecule-1" OR “VCAM-1” OR “Vascular Cell Adhesion Molecule 1” OR “Intercellular Adhesion Molecule-1“ OR “ICAM-1” OR fibrinogen OR "Tumor Necrosis Factor-alpha" OR "TNF-alpha" OR "TNF alpha" OR “Serum Amyloid A Protein”)
**Body composition variables**	(“body mass index” OR “BMI” OR “Body Weight” OR “Body Composition” OR Anthropometry OR “waist circumference” OR Adiposity OR obesity OR “blood pressure”)
