# Supplementary material for: Circulating microRNA Related to Cardiometabolic Risk Factors for Metabolic Syndrome: A Systematic Review
Source: Metabolites. 2022 Oct 30;12(11):1044. doi: 10.3390/metabo12111044 (PMC9692352; doi:10.3390/metabo12111044)
Supplement: Supplementary file 1 [file metabolites-12-01044-s001.zip › Table S2_Papers excluded stage 2_Supplementary_edited.pdf]

Table S2. Papers excluded after full reading and reasons for exclusion.

Author and year of study	Title	Reasons for papers exclusion
Noren-Hooten et al. 2013	Age-related changes in microRNA levels in serum	Article did not assess correlations between microRNA and variables of glucose and lipid metabolism or blood pressure or inflammation
Olivieri et al. 2012	Age-related differences in the expression of circulating microRNAs: miR-21 as a new circulating marker of inflammaging	Article did not assess correlations between microRNA and variables of glucose and lipid metabolism or blood pressure or inflammation
Braga 2018	Análise de miRNoma em sangue periférico de indivíduos com obesidade e resistência à insulina TT	Thesis
Ashoori et al. 2018	Apelin-13 serum levels in type 2 diabetic obese women: Possible relations with microRNAs-107 and 375	Article did not assess correlations between microRNA and variables of glucose and lipid metabolism or blood pressure or inflammation
Jiménez-Lucena et al. 2018	A plasma circulating miRNAs profile predicts type 2 diabetes mellitus and prediabetes: from the CORDIOPREV study	Individuals had established coronary health disease
Knoka et al. 2019	Association between circulating microRNAs and metabolic biomarkers in patients with prediabetes with or without early atherosclerosis	Work presented in conference as abstract and sample composed by individuals with coronary artery disease
Munetsuna et al. 2018	Association of subcutaneous and visceral fat with circulating microRNAs in a middle-aged Japanese population	The article have not control group
Ameling et al. 2015	Associations of circulating plasma microRNAs with age, body mass index and sex in a population-based study	The article have not control group
Alramah et al. 2019	Associations of targeted circulating microRNAs with insulin resistance in the camera (carotid atherosclerosis: Metformin for insulin resistance) trial	Work presented in conference as abstract and sample composed by individuals with coronary artery disease
Spinetti et al. 2016	Blood circulating microRNA-15a and microRNA-16 in diabetic patients: Preliminary results of the DIAPASON study	Work presented in conference as abstract
Raitoharju et al. 2016	Blood hsa-miR-122-5p and hsa-miR-885-5p levels associate with fatty liver and related lipoprotein metabolism-The Young Finns Study	Follow-up study and microRNA assessed in whole blood
Raitoharju et al. 2014	Blood microRNA profile associates with the levels of serum lipids and metabolites associated with glucose metabolism and insulin resistance and pinpoints pathways underlying metabolic syndrome: the cardiovascular risk in Young Finns Study	microRNA assessed in whole blood
Fejes et al. 2015	Changes in the expression of plasma and platelet microRNAs in type 2 diabetes mellitus and obesity	Work presented in conference as abstract
Willeit	Circulating microRNA-122 is associated with incident metabolic syndrome and type-2-diabetes	Work presented in conference as abstract

et al. 2015		
Willeit et al. 2017	Circulating MicroRNA-122 Is Associated With the Risk of New-Onset Metabolic Syndrome and Type 2 Diabetes	Cohort study and sample had individuals with cardiovascular disease
Wen; Qiao; Wang 2015	Circulating microRNA-223 as a potential biomarker for obesity	Case-control study in which individuals received lifestyle intervention

(continues)

(continuation)

Author and year of study	Title	Reasons for papers exclusion
Sodi et al. 2015	Circulating microRNA-30c is associated with total-and LDL-cholesterol	Work presented in conference as abstract
Norizam et al. 2014	Circulating microRNA as biomarkers for young dyslipidemia men	Work presented in conference as abstract
Bajomo et al. 2013	Circulating MicroRNA in the assessment of obesity	Work presented in conference as abstract
Karolina; Armugam; Jeyaseelan 2011	Circulating micrornas: Potential biomarkers in artherosclerosis	Work presented in conference as abstract
Jaeger et al. 2018	Circulating microRNAs-192 and-194 are associated with the presence and incidence of diabetes mellitus	Clinical discovery and validation studies
Flowers et al. 2019	Circulating microRNAs are associated with glycemic variability over a 12-month period	Paper not retrieved
Iacomino et al. 2016	Circulating microRNAs are deregulated in overweight/obese children: Preliminary results of the I. Family study	Article did not assess correlations between microRNA and variables of glucose and lipid metabolism or blood pressure or inflammation
Khalyfa et al. 2016	Circulating microRNAs as Potential Biomarkers of Endothelial Dysfunction in Obese Children	Individuals had endothelial dysfunction
Ma et al. 2015	Circulating microRNA species and role in human insulin resistance	Work presented in conference as abstract
Zhang; Shang; Wang 2015	Circulating miR-126 is a potential biomarker to predict the onset of type 2 diabetes mellitus in susceptible individuals	Follow-up study
Yildrim et al. 2019	Circulating miR-21 levels in hypertensive patients with asymptomatic organ damage	Individuals had asymptomatic organ damage
Miao et al. 2019	Circulating miR-3659 may be a potential biomarker of dyslipidemia in patients with obesity	Article used integrated bioinformatic methods

Karolina et al. 2012	Circulating miRNA profiles in patients with metabolic syndrome	MicroRNA quantified in blood and exosomes
Iacomino et al. 2015	Circulating miRNAS are deregulated in overweight/obese children: Preliminary results of the I. family study	Work presented in conference as abstract
Niemina et al. 2018	Circulating miRNAs as predictive biomarkers for type 2 diabetes development in individuals at risk: Outcomes of a 5-year prospective observational cohort study	Work presented in conference as abstract
Camargo et al. 2018	Circulating miRNAs as predictive biomarkers of type 2 diabetes mellitus development: From the CORDIOPREV study	Work presented in conference as abstract

(continues)

(continuation)

Author and year of study	Title	Reasons for papers exclusion
Khalyfa et al. 2015	Circulating mirnas-potential biomarkers of endothelial dysfunction in children	Work presented in conference as abstract
Chen et al. 2019	Correlation between serum microRNA-122 and insulin resistance in obese children	Paper written in Chinese
Wang et al. 2014	Determination of 14 circulating microRNAs in Swedes and Iraqis with and without diabetes mellitus type 2	The article have not control group
Guo et al. 2019	Diagnostic Value of Serum MiR-587 in Patients with Metabolic Syndrome	Paper not retrieved
Yan et al. 2016	Differential expression of microRNAs in plasma of patients with prediabetes and newly diagnosed type 2 diabetes	Article did not assess correlations between microRNA and variables of glucose and lipid metabolism or blood pressure or inflammation
Zandoná et al. 2016	Differential plasma concentrations of MicroRNA-16-5p and MicroRNA-19b in Brazilian lean and obese children	Work presented in conference as abstract
Lin et al. 2015	Elevated circulating microRNA-143-3p is associated with insulin resistance in the metabolic syndrome	Work presented in conference as abstract
Thompson et al. 2017	Elevation of circulating microRNA levels in obese children compared to healthy controls	Individuals had non-alcoholic fatty liver disease
Azmir et al. 2014	Identification of circulating microRNAs in young men with central obesity	Work presented in conference as abstract
Ding et al. 2016	Identification of the differential expression of serum microRNA in type 2 diabetes	Article did not assess correlations between microRNA and variables of glucose and lipid metabolism or blood pressure or inflammation
Kilic et al. 2015	microRNA-143 and-223 in obesity	microRNA assessed in whole blood

Author and year of study	Title	Reasons for papers exclusion
Dias et al. 2017	MicroRNA expression profiling for the early detection of type 2 diabetes in South African women of mixed ethnic ancestry	Work presented in conference as abstract
Masotti et al. 2017	Oral glucose tolerance test unravels circulating miRNAs associated with insulin resistance in obese preschoolers	Age < 5 years old
Gao et al. 2012	Plasma levels of lipometabolism-related miR-122 and miR-370 are increased in patients with hyperlipidemia and associated with coronary artery disease	Individuals had coronary artery disease
Trusinskis et al. 2017	Plasma microRNA-155 predicts increased plaque vulnerability in patients with prediabetes	Work presented in conference as abstract
Iacomino et al. 2019	Plasma microRNA expression profiles are associated with early childhood obesity: Results of the I.Family Study	Work presented in conference as abstract
Zampetaki et al. 2010	Plasma MicroRNA profiling reveals loss of endothelial MiR-126 and other MicroRNAs in type 2 diabetes	Validation study. Article did not assess correlations between microRNA and variables of glucose and lipid metabolism or blood pressure or inflammation
Zhao et al. 2017	Plasma MicroRNA signature predicting weight gain among Mexican-American women	Follow-up study
Zhang et al. 2013	Plasma miR-126 Is a Potential Biomarker for Early Prediction of Type 2 Diabetes Mellitus in Susceptible Individuals	Article did not assess correlations between microRNA and variables of glucose and lipid metabolism or blood pressure or inflammation
Flowers et al. 2017	Preliminary evidence supports circulating microRNAs as prognostic biomarkers for type 2 diabetes	The sample was composed of participants from clinical trials that studied behavioral risk reduction interventions
Ma; Fu; Garvey 2018	Relationship of Circulating miRNAs with Insulin Sensitivity and Associated Metabolic Risk Factors in Humans	The article have not control group
Rezk; Sabbah; Saad 2016	Role of microRNA 126 in screening, diagnosis, and prognosis of diabetic patients in Egypt	Individuals had diabetic macrovascular complications, nephropathy,retinopathy,and neuropathy
Pescador et al. 2013	Serum Circulating microRNA Profiling for Identification of Potential Type 2 Diabetes and Obesity Biomarkers	Article did not assess correlations between microRNA and variables of glucose and lipid metabolism or blood pressure or inflammation
Wang et al. 2013	Serum Microrna 122 levels correlate positively with alanine aminotransferase levels and insulin resistance in Chinese young adults	Paper not retrieved

(continues)
(continuation)

Yang et al. 2017	Serum microRNA profiling and bioinformatics analysis of patients with type 2 diabetes mellitus in a Chinese population	Article did not assess correlations between microRNA and variables of glucose and lipid metabolism or blood pressure or inflammation
Yang et al. 2014	Serum miR-23a, a potential biomarker for diagnosis of pre-diabetes and type 2 diabetes	Validation study. Article did not assess correlations between microRNA and variables of glucose and lipid metabolism or blood pressure or inflammation
Xu et al. 2019	Several circulating miRNAs related to hyperlipidemia and atherosclerotic cardiovascular diseases	Individuals had atherosclerotic cardiovascular diseases
Meerson et al. 2019	Sex Differences in Plasma MicroRNA Biomarkers of Early and Complicated Diabetes Mellitus in Israeli Arab and Jewish Patients	Individuals had renal and vascular complications
Kong et al. 2011	Significance of serum microRNAs in pre-diabetes and newly diagnosed type 2 diabetes: A clinical study	Validation study. Article did not assess correlations between microRNA and variables of glucose and lipid metabolism or blood pressure or inflammation
Prats-Puig et al. 2013	Study of circulating microRNAs in prepubertal obesity	Work presented in conference as abstract
Eastwood; Caslake; Sodi 2016	The association of circulating microRNA-30c with atherogenic lipoprotein subfractions and composition	Study determined association with atherogenic lipoprotein subfractions
Chuan et al. 2015	The changes in miR-130b levels in human serum and the correlation with the severity of diabetic nephropathy	Individuals had diabetic nephropathy

(continues)

(continuation)

**Author and year of study**	**Title**	**Reasons for papers exclusion**
Liang et al. 2014	The circulating miRNAs expression in simple obese children	Work presented in conference as abstract
Zhang et al. 2018	The diagnostic values of circulating miRNAs for hypertension and bioinformatics analysis	Validation study with bioinformatics analysis
Corona-Meraz et al. 2019	The increase of circulating miR-143 and miR-33B relative expression, and adiponectin multimeric forms dysregulation are associated with the body fat storage and immunometabolic profile in individuals with obesogenic phenotype	Work presented in conference as abstract
Can; Buyukinan; Yerlikaya 2016	The investigation of circulating microRNAs associated with lipid metabolism in childhood obesity	microRNA assessed in whole blood
Eastwood et al. 2016	The relationship between circulating microRNA and lipid indices	Work presented in conference as abstract

Liu et al. 2014	The role of circulating microRNA-126 (miR-126): A novel biomarker for screening prediabetes and newly diagnosed type 2 diabetes mellitus	Individuals had diet and physical activity intervention
Al-Muhtaresh; Salem; Al-Kafaji 2019	Upregulation of Circulating Cardiomyocyte-Enriched miR-1 and miR-133 Associate with the Risk of Coronary Artery Disease in Type 2 Diabetes Patients and Serve as Potential Biomarkers	microRNA assessed in whole blood
Beeghly-Fadiel et al. 2013	Variation in circulating miRNA across physiologic traits: Implications for biomarker studies	Work presented in conference as abstract
Mononen et al. 2019	Whole blood microRNA levels associate with glycemic status and correlate with target mRNAs in pathways important to type 2 diabetes	microRNA assessed in whole blood
Kimura et al. 2016	Clinical Significance of Determining Plasma MicroRNA33b in Type 2 Diabetic Patients with Dyslipidemia	Study did not have a healthy control group and individuals had nutritional advice to control diabetes
Doumatey et al.2018	Circulating MiR-374a-5p is a potential modulator of the inflammatory process in obesity	Study did not have a healthy control group
